# Effects of Resveratrol against Lung Cancer: In Vitro and In Vivo Studies

**DOI:** 10.3390/nu9111231

**Published:** 2017-11-10

**Authors:** Michael Yousef, Ioannis A. Vlachogiannis, Evangelia Tsiani

**Affiliations:** 1 Department of Health Sciences, Brock University, St. Catharines, ON L2S 3A1, Canada; my11dq@brocku.ca; 2 Centre for Bone and Muscle Health, Brock University, St. Catharines, ON L2S 3A1, Canada; yv17av@brocku.ca

**Keywords:** resveratrol, lung cancer, proliferation, survival, cell signaling

## Abstract

Uncontrolled cell growth and resistance to apoptosis characterize cancer cells. These two main features are initiated in cancer cells through mutations in key signaling molecules, which regulate pathways that are directly involved in controlling cell proliferation and apoptosis. Resveratrol (RSV), a naturally occurring plant polyphenol, has been shown to have biological effects counteracting different diseases. It has been found to provide cardio-protective, neuro-protective, immuno-modulatory, and anti-cancer health benefits. RSV has been found to inhibit cancer cell proliferation, induce cell cycle arrest and apoptosis, and these anticancer effects may be due to its ability to modulate signaling molecules involved in these processes. The present review summarizes the existing in vitro and in vivo studies on resveratrol and its anti-lung cancer properties.

## 1. Introduction

Cancer cells are most commonly identified by their high rate of proliferation and resistance to programmed cell death/apoptosis [1]. Cancer cells are self-sufficient in growth signals, and insensitive to anti-growth signals. Their cell replication is up regulated while their programmed cell-death pathway (apoptosis) is down regulated. They have sustained angiogenesis, ensuring that the cells are vascularized by blood vessels and they have an ability to invade tissues and metastasize [1]. 

Lung cancer is a malignant lung tumor characterized by uncontrolled cell growth in lung tissue. It is the most common cancer among men in both incidence and mortality. Among women, lung cancer is the third highest in incidence and second in mortality after breast cancer. In 2012, there were 1.82 million new cases and 1.56 million deaths globally due to lung cancer, representing approximately 19.4% of all deaths from all types of cancer [2].

Plant derived compounds and bioactive food components have recently emerged as agents with anticancer properties [3,4,5,6]. One such bio-molecule is resveratrol (trans-3,5,4′-trihydroxystilbene) [3]. Resveratrol (RSV) is a naturally occurring polyphenol that is most commonly found in grapes, red wine, and peanuts [7]. Specifically, grape skin contains 50–100 mg of RSV per gram [8] but the dominant source of RSV is the root of *Polygonum cuspidatum,* which is widely used in traditional Chinese and Japanese medicine [9,10]. The RSV concentration in red wine is significantly higher than the respective concentration in white wine, a fact that is attributed to the differences in the wine fermentation processes. The average concentration or RSV in red wine ranges from 0.84 to 7.33 mg/L [11], while, in white wine, ranges from 0.01 to 0.08 mg/L [12,13].

Resveratrol has been found to have health benefits such as protection against cardiovascular disease, aging, metabolic disease and cancer [7]. The most known property of RSV is its antioxidant effect which allows it to convert free radical like reactive oxygen species (ROS) to unreactive compounds [14]. A groundbreaking study by Jang et al. found that resveratrol has anticancer properties and since then numerous in vitro and in vivo studies have been performed [15] and other studies showed that acts as a cancer chemopreventive too [16].

Over the years, numerous reviews have been published focusing on the anticancer effects of RSV ([15,17,18,19,20,21]. However, the present review highlights the current in vitro and in vivo studies that have been performed examining the effects of resveratrol and its derivatives on lung cancer.

## 2. Effects of Resveratrol (RSV) as a Single Agent against Lung Cancer: In Vitro Studies

Resveratrol (RSV) has been studied as a single agent treatment in various lung cancer cells (Table 1). A549 and H460 cell treatment with RSV inhibited growth, up-regulated microtubule-associated protein 1 light chain 3 (LC3) and increased Proline-, glutamic acid–, and leucine-rich protein-1(PELP1) accumulation in autophagosomes with GFP-LC3 [22]. A549 cell treatment with RSV induced apoptosis and G1 cell cycle arrest. It was also found that there was an up-regulation of p53 and p21, an increased activation of caspases, disruption of the mitochondrial membrane complex and an altered expression of cyclin A, chk1, CDC27 and Eg5 [23]. These effects were found to be mediated through the TGF-β/Smad pathway. RSV treatment also showed a down-regulation of Smad activators 2 and 4 and up-regulation of repressor Smad 7 [23]. A549, A427 and NCI-H23 cells treated with RSV showed an inhibition of the phosphatidylinositol-3-kinase (PI3K) pathway and decreased mTOR phosphorylation [24]. Interestingly, BEAS-2B cell treatment with 2,3,7,8-tetrachlorodibenzo-p-dioxin (TCDD) and RSV repressed TCDD-inducible gene transcription in estrogen receptor (ER+) human lung cancer [13]. Specifically it was found that RSV completely abrogated TCDD-induced CYP1A1 gene transcription [25]. Wu et al. reported that A549 cell treatment with 4′-chloro-3,5-dihydroxystil bene (DHS), an analog of resveratrol, increased ROS levels, induced cell cycle arrest, decreased mitochondrial membrane potential, decreased Poly (ADP-ribose) polymerase degradation, increased intracellular acidic vacuoles and increased LC3-II formation and intracellular GFP-LC3 aggregation all of which resulted in a decrease in cell proliferation [26]. SPC-A-1 cells treated with RSV had reduced proliferation and increased apoptosis and cell cycle arrest [26]. Zhao et al. reported that these effects were mediated through an activation of caspase-3 and reduced survivin levels [27]. Treatment of A549 cells with RSV caused a 2-ford alteration on 71 micro-RNA (miRNA) expression [28]. SPC-A-1 cells treated with RSV had a reduction in proliferation and an increase in apoptosis and cell cycle arrest. H1299 cell treatment with RSV resulted in an inhibition of glycolysis mediated through a reduction of histone H2B mono-ubiquitination [29]. Han et al. reported that 16HBE-T and H460 cell treatment with RSV resulted in decreased cell viability and cell proliferation as well as initiated G0 cell cycle arrest. Up-regulation of miR-622 expression in A549 cells was also observed and it was found that miR-622 targets K-Ras downstream resulting in inhibition of proliferation [30]. Trimethoxyl Stilbene (TMS) (an analog of RSV) treatment of A549 cells resulted in inhibition of proliferation and induction of apoptosis in a dose-dependent manner [19]. It was also found that treatment with TMS up-regulated caspase-3 expression and cleavage, up-regulated IκB and down-regulated NF-κB, STAT3, STAT5b and JAK2 [31]. ASTC-A-1 cells treated with RSV had an increased rate of apoptosis and caspase-3 and -9 expression [32]. A549, H1299 and H460 cells treated with RSV showed reduced cell viability in a dose-dependent manner, which was also found to be p53 dependent [21]. Caspase-9 and -7 activation and PARP-cleavage were found to be increased with RSV treatment [21]. It was also found that transient transfection with WT p53-GFP gene caused H1299 cells to become more responsive to the apoptotic properties of RSV [33]. A549 cell treatment with pterostilbene (an analog of RSV) inhibited cell growth, stimulated apoptosis and autophagosome accumulation as well as lysosomal membrane permeabilization [34]. Jung et al. found that RSV treatment of LLC cells suppressed 18F-FDG uptake [23]. It was also found that glycose influx and Glut-1 expression were reduced, HIF-1α expression and Akt activation were decreased, and ROS levels were increased [35]. A549 and H1299 cells treated with RSV were found to exhibit a dose-dependent inhibition of growth, with no effect on expression of cleaved PARP and activated caspase-3. This suggests that low-dose RSV treatment inhibits growth in an apoptosis-independent mechanism. To test this theory, other mechanisms of growth inhibition were studied. It was found that SA-β-gal expression was increased, p53 and p21 expression was increased, double-stranded DNA breaks were increased, ROS levels were elevated, Nox5 expression was up-regulated, and EF1A expression was decreased [36]. A549 cells pre-treated with Benzo(a)pyrene followed by RSV treatment, resulted in a decrease in cell viability, an increase in p53 levels, G2/M cell cycle arrest and an induction of apoptosis. Bcl-2 down-regulation, decreased cyclin D expression, down-regulated NF-κB and Iκκ1 expression, increased p21 expression and increased TRAIL receptors 1 and 2 expression [37]. A549 cells treated with RSV showed decreased cell proliferation, inhibition of TGF-β1 induced epithelial to mesenchymal transition (EMT) and suppression of cell adhesion [26]. Dose-dependent cell growth inhibition and activation of caspase-3 of A549 cells was observed with RSV treatment [38]. RSV treatment of CL-1-5, A549, H322 and H1435 cells resulted in inhibition of growth/proliferation, down-regulated Akt, IκB and NF-κB [39]. TMS (an analog of RSV) treatment of H1975 cells increased intracellular calcium levels in Gefitinib resistant NSCLC. TMS treatment was found to only induce anti-proliferative effects in Gefitinib resistant NSCLC, not normal NSCLC and normal lung epithelial cells. This was associated with decreased EGFR phosphorylation and activation, induction of caspase-independent apoptosis and autophagy by direct binding to SERCA and inducing ER stress and AMPK activation, suppression of the mTOR pathway and increased JNK activity [40]. Lucas et al. showed that A549 cells treated with RSV reduced growth mediated through an induction of caspase-3 activity [41]. Treatment with RSV conjugated nanoparticles (NP) followed by cigarette smoke condensate (CSC) showed that RSV alone attenuated CSC-induced DNA fragmentation in H727 cells. However, NPs dramatically increased RSV induced apoptosis in CSC-treated cells. These results indicate that NPs are capable of increasing the efficacy of lipophilic drugs such as RSV [42]. A549 cells treated with RSV exhibited a decrease in cell proliferation, cell viability and cell cycle arrest that was associated with reduced AK001796 non-coding RNA [43]. A549 cells treated with RSV showed reduced proliferation as well as G0/G1 cell cycle arrest, increased in p53 nuclear expression, down-regulation of cyclin D1, CDK4 and CDK6 expression as well as an up-regulation of the CDK inhibitors, p21 and p27 [44]. A549 cells treated with RSV showed reduced P62 levels, increased p62 degradation and an increase in Fas/Cav1 complex formation [20]. Fas/Cav1 activated complexes lead to an increase in caspase-8 mediated Beclin-1 cleavage, resulting in c-terminal Beclin-1 translocation to the mitochondria to initiate apoptosis [32]. A549 cells treated with THS (an analog of RSV) showed a dose-dependent induction of apoptosis and autophagy. Up-regulation of cleaved PARP, increased caspase-3 and -9, increased LC3-II accumulation, down-regulation of Bcl-2, inhibition of the mTOR pathway and elevated ROS levels were also observed in these cells [45]. A549 cells treated with RSV-loaded nanoparticles had reduced H_2_O_2_ induced ROS levels, increased RSV uptake, activated Nrf2-Keap1 signaling and accumulation of Nrf2 [46]. A significant inhibition of LLC cell growth was seen with treatment with DHS (an analog of RSV). It was also accompanied by a decrease in LLC cell migration and invasion [47]. CEM and A549 cells treated with RSV showed a decrease in proliferation, an increase in apoptosis, an inhibition of tubulin polymerization, G2/M cell cycle arrest, loss of the mitochondrial membrane potential as well as activation of caspase-3 and -9 and PARP cleavage [48].

## 3. Effects of Resveratrol (RSV) in a Combination Treatment against Lung Cancer: In Vitro Studies

There have also been several in vitro studies looking at the effect of RSV as part of a combination treatment (Table 2). These studies are summarized in the following section. A549 and H460 cells pre-treated with RSV followed by Gy IR treatment resulted in a synergistic enhancement of the IR-induced cell killing in NSCLC through an apoptosis-independent mechanism. This apoptosis-independent mechanism was found to be increased percentage of SA-β-gal positive senescent cells and an increase in double-stranded DNA breaks [52]. H-2452 cells treated with a combination of RSV and Clofarabine showed a synergistic decrease in Msl-1 protein expression with little effect on Bcl-xL expression [41]. However, it was found that Bcl-xL knockout enhanced the ability of the combination treatment to inhibit cell proliferation and increase apoptosis [41]. The increase in apoptosis found through the combination treatment of these cells was found to be mediated through G2/M phase cell cycle arrest and increased caspase-3 and -7 activity as well as an increase in caspase-3 cleavage [53]. H460, A549, PC-9 and H1975 cell treatment with a combination of RSV and Erlotinib resulted in a synergistic enhancement of Erlotinib-induced apoptosis, reduced cell viability and colony formation [42]. An increase in ROS production, reduction in expression of anti-apoptotic proteins, such as survivin and Mcl-1, promotion of p53 and PUMA expression, increased caspase-3 activity was observed and the combination was more effective at inhibiting the Akt/mTOR/p70s6K pathway [54]. A549 cells grown as spheroid bodies to resemble cancer stem cells (CSCs) were transfected with the ZD55 oncolytic adenovirus and treated with RSV in a study by Yang et al. [39]. The ZD55 virus was carrying the TRAIL gene (ZD55-TRAIL). ZD55-TRAIL alone induced cytotoxicity, however, the combination treatment increased the ZD55-TRAIL mediated cytotoxicity [39]. It was also found that the induction of apoptosis in these cells was caspase dependent with decreased levels of pro-caspase-9, -8 and -3 [51]. Zhu et al. showed that PC9/G treatment with Gefitinib and RSV resulted in a synergistic inhibition of proliferation of Gefitinib-resistant NSCLC cells [43]. Co-treatment also induced apoptosis, autophagy, cell cycle arrest and senescence [43]. These effects were found to be mediated through an inhibition of EGFR phosphorylation by increasing Gefitinib accumulation intracellularly, increased CYP1A1 and ABCG2 expression and increased expression of cleaved caspase-3, LC3B-II, p53 and p21 [55]. Finally, Lung fibroblasts affected with idiopathic pulmonary fibrosis pre-treated with RSV followed by TGF-β or CXCL12 showed that RSV is capable of repressing fibroblast to myofibroblast conversion in lung cells. It was also shown that RSV is capable of reversing fibroblast to myofibroblast conversion in lung cells if they were treated with TGF-β and CXCL12 followed by RSV [56].

## 4. Effects of Resveratrol (RSV) in Lung Cancer: In Vivo Studies

Few studies have looked at the effect of resveratrol treatment of lung cancer in vivo (Table 3). Female nude mice (five weeks old) injected subcutaneously with A549 cells were treated with 50 mg/kg DHS, an analog of resveratrol, intraperitoneally from Day 1 to Day 4 and then Day 7 to Day 10. This treatment regimen resulted in a decrease in tumor growth [26]. Zhao et al. reported that 18 female Balb/c mice injected subcutaneously with SPC-A-1 cells into their flanks showed an inhibition of tumor growth upon treatment with 1 or 3 g/kg/day RSV for 28 days in their diet [27]. Nude mice inoculated with A549 cells were treated with 20 mg/kg RSV every other day for 25 days, resulted in an inhibition of metastasis and activation of SIRT1 [57]. Nude mice subcutaneously injected with A549 cells were treated with 15, 30 or 60 mg/kg RSV injections for 15 days in a study by Yin et al., which found that RSV inhibits lung cancer growth in a dose-dependent manner [38]. Male mice given 100 mg/kg body weight Benzo(a)pyrene (BP) to induce lung carcinogenesis were treated with 5.7 μg/mL RSV in drinking water and 60 mg/kg body weight curcumin for 22 weeks. The BP-treated mice showed increased levels of p53 hyper-phosphorylation and decreased activity of caspase-3 and -9. The combination treatment lead to an increase in caspase-3 and -9 activity and reduced p53 hyper-phosphorylation, which completely counteracted the activity of BP treatment [58]. Finally, Savio et al., studied the effect of 25 mg/kg/day DHS in drinking water for seven days on sixty male C57B6 mice (four weeks old) bearing LLC tumors, and a decrease in tumor volume, cell proliferation, tumor angiogenesis and liver metastatic lesions was observed [47]. LLC tumor cells were injected subcutaneously into the flanks of C57BL/6 mice and treated with RSV from Day 10 after the injection until their sacrifice (four weeks after injection). RSV treatment led to an inhibition of lung cancer tumor growth through reduction of F4/80+ expressing cells and M2 polarization in tumors [59].

## 5. Molecular Targets/Signaling Molecules Contributing to Anticancer Effects of RSV

Cancer cells overexpress growth factor receptors, such as the epidermal growth factor receptor (EGFR) [60] and glucose transporters [61] giving them advantages towards proliferation and survival. Binding of growth factors to their respective receptors enhances the intrinsic tyrosine kinase activity of the receptor and activates signaling pathways, including the phosphoinositide-3-kinase (PI3K)/Akt/mammalian target of rapamycin (mTOR) pathway, that leads to increased proliferation and inhibition of apoptosis [62]. Activation of the growth factor receptors also leads to activation of the Ras-mitogen activated protein kinase (Ras-MAPK) cascade, resulting in enhanced proliferation [63]. On the other hand, the pathway of p53, a well-established tumor suppressor, is often found down-regulated, if not silenced, in many types of cancer cells [50].

Evidence from all the studies presented in the current review indicate that RSV in lung cancer cells acts as an inhibitor of EGFR [40,55], mTOR [24,40], and Akt [35,39,50]. In addition, the NF-κB [31,37,39] and the JAK/STAT [31] pathway and the GLUT 1 glucose transporter [35] were inhibited by RSV. On the other hand, RSV activated the tumor suppressor p53 [23,35,44,50,52,54,55,58] and increased caspase activity [23,27,31,32,33,38,40,41,45,48,54,55,58] orienting the cancer cells towards an apoptotic pathway. Furthermore, RSV has been established as an activator of SIRT1, a histone deacetylase that plays important role in many fundamental cellular processes [64,65]. Sun et al. found a significant increase of SIRT1 activation in A549 lung cancer cells treated with RSV [57]. Importantly, in colon cancer cells, RSV resulted in decrease of NF-κB activation [66] by a mechanism that was mediated by SIRT1 activation [67]. 

## 6. Conclusions 

Cancer is defined by a high rate of mutation often inducing chemotherapeutic resistance to established treatments and, in recent years, there has been an increasing focus on finding new cancer therapies. From the studies summarized in this review, it is evident that resveratrol is capable of: (1) decreasing cancer cell proliferation and inhibiting tumor growth; (2) inducing cell cycle arrest; (3) inducing cell apoptosis; and (4) inhibiting metastasis of lung cancer. In many studies, RSV was found to enhance ROS production in cancer cells inducing cytotoxicity.

Resveratrol has low bioavailability and the plasma resveratrol concentrations have been reported to be at nanomolar or lower levels. Most of the studies presented here used concentrations in the μM range, which may not accurately represent physiologically relevant concentrations; however, one study showed that resveratrol conjugated NPs increased the activity of resveratrol and enhanced its uptake by cells. This poses a promising and novel area of research to help increase uptake of lipophilic drugs such as resveratrol, which in general tend to have very low bioavailability. Further research should be conducted on resveratrol bioavailability and resveratrol metabolites to understand the mechanism behind the resveratrol induced anti-cancer effects. Resveratrol was also shown through numerous studies presented here to not only act synergistically with chemotherapeutic drugs to increase their anti-cancer effects, but it was also shown to reverse resistance to those chemotherapeutics, which is very desirable as the prevalence of chemotherapy resistant cancer cells increases. All together, these findings present resveratrol as a promising anti-cancer treatment and warrant further studies to better understand the mechanism behind resveratrol induced anti-cancer effects.

## Figures and Tables

**Table 1:** Effects of resveratrol (RSV) as a single agent against lung cancer: in vitro studies.

Cancer Cell	Dose/Duration	Findings	Mechanism	Reference
A549 and H460	100 μM RSV for 24 h	↓growth	↑LC3 ↑PELP1 accumulation in autophagosomes with GFP-LC3	[22]
A549	25, 50, 100 μM RSV for 48, 72, 96 h	↑apoptosis ↑cell cycle arrest	↑p53 and p21 ↑caspases ↑disruption of the mitochondrial membrane complex G1 cell cycle arrest Altered expression of cyclin A, chk1, CDC27 and Eg5 ↓Smad activators 2 and 4 ↑repressor Smad 7	[23]
A549, A427 and NCI-H23	20, 50, 100 μM RSV for 2, 4 or 8 h	↓PI3K pathway ↓tumor formation	↓mTOR phosphorylation	[24]
BEAS-2B	2,3,7,8-tetrachlorodibenzo-p-dioxin (TCDD) + 0.01, 0.05, 0.1, 0.5, 1, 5, 10 μM RSV for 6 h	RSV is a potent repressor of TCDD inducible gene transcription in estrogen receptor (ER+) human lung cancer	RSV completely abrogates TCDD-induced CYP1A1 gene transcription	[25]
A549	12.5, 25, 37.5, 50, 62.5, 75, 87.5, 100 μM DHS for 48 h	↓cell proliferation	↑ROS species Sub G1 formation (cell cycle arrest) ↓mitochondrial membrane potential ↓Poly (ADP-ribose) polymerase degradation ↑intracellular acidic vacuoles ↑LC3-II formation and intracellular GFP-LC3 aggregation	[26]
SPC-A-1	25 μM, 50 μM or 100 μM RSV, for up to 96 h	↓proliferation ↑apoptosis ↑cell cycle arrest	↑caspase-3 ↓survivin levels	[27]
A549	60, 120 μM RSV for 24 h	Altered miRNA expression (miRNA is involved in initiating lung cancer)	-	[28]
H1299	10 mM–500 mM RSV, for 4 h	↓glycolysis	↓mono-ubiquitination of histone H2B	[29]
16HBE-T and H460	12.5, 25, 50 μM RSV for 48 h	↓cell viability ↓cell proliferation. ↑cell cycle arrest	↑miR-622 expression ↑G0 cell cycle arrest K-ras is downstream target of miR-622	[30]
A549	1.25, 2.5, 5, 7.5, 10 μM Trimethoxyl Stilbene (TMS) for 48 h	TMS inhibited proliferation and induced apoptosis in a dose-dependent manner	↑Up-regulation and cleavage of caspase-3 ↑IKB ↓NF-κB, STAT3, STAT5b and JAK2 signal transduction	[31]
ASTC-A-1	0 μM–125 μM RSV for 48 h	Induction of apoptosis	↑caspase-3 and-9	[32]
A549, H1299 and H460	100, 200, 300, 400, 500 μM RSV for 5 min, 24, 48 h	↓cell viability (p53 dependent) Transient transfection of WT p53-GFP gene caused H1299 cells to become more responsive to the pro-apoptotic properties of RSV	↑caspase-9 and -7 activation ↑PARP cleavage	[33]
A549	20, 40, 60, 80, 100 μM Pterostilbene for 48 h	↓cell growth. ↑apoptosis. Autophagosome accumulation Lysosomal membrane permeabilization	HSP70 protein deficiency showed high susceptibility to pterostilbene.	[34]
LLC	50 μM RSV for 24 h	↓18F-FDG uptake	↓glycolytic flux and Glut-1 expression ↑ROS ↓HIF-1a expression ↓Akt activation	[35]
A549 and H460	10, 20, 50 μM RSV for 10 to 12 days	Inhibition of growth in a dose-dependent manner. No effect on expression of cleaved PARP and activated caspase-3, suggesting that low dose RSV treatment inhibits growth in an apoptosis-independent mechanism	(1) Increase in SA-B-gal (2) Increased p53 and p21 expression (3) Decreased EF1A expression (4) Increased double-stranded DNA breaks (5) Increased ROS (6) Upregulated Nox5 expression	[36]
A549	20 μM Benzo(a)pyrene for 48 h pre-treatment + 10 μM RSV for 24 h	Decreased cell viability. Increased p53 levels. Cell cycle arrest. Apoptosis	(1) Down-regulation of Bcl-2 expression (2) Decreased cyclin D expression (3) Increased p21 expression (4) Increased TRAIL receptors 1 and 2 expression (5) Down-regulation of NF-KB and IKK1 expression (6) Induction of G2/M cell cycle arrest	[37]
A549	0 μM–40 μM RSV, for 48 h	Decreased proliferation and EMT. Suppression of cell adhesion	Inhibition of the morphological changes of TGF-β1 induced EMT.	[49]
A549	2, 4, 8, 16, 32, 64 μM RSV for 48 h	RSV exerts dose-dependent cell inhibition	Activation of caspase-3	[38]
CL1-5, A549, H322 and H1435	20 μM RSV, for 48 h	Suppression of tumor growth	Downregulation of Akt, I-κB and NF-κB	[39]
A549 and H1299	0.02, 2% red wine (equivalent to 4, 400 nM RSV) and 0.5, 2% white wine	Inhibition of cell proliferation. Wine mixture induced effects that were only reproducible at 50 μM RSV treatment alone	(1) Reduced basal and EGF-stimulated Akt and Erk phosphorylation (2) Increased p53 expression and phosphorylation	[50]
H1975	20, 40, 60, 80 nM TMS for 24 h	Elevated intracellular calcium levels in Gef resistant NSCLC. Anti-proliferative effect only in G-R NSCLC but not normal NSCLC and normal lung epithelial cells	(1) Decreased EGFR phosphorylation and activation (2) Induction of caspase-independent apoptosis and autophagy by directly binding to SERCA and causing ER stress and AMPK activation (3) Suppressed the mTOR pathway (4) Increased JNK activity	[40]
A549	5.5 μM–175.2 μM RSV, for 24h	Inhibition of growth	Induction of caspase-3	[41]
A549	0.05, 0.10, 0.23 μM RSV + 8.14 μg/mL NP for 24 h pre-treatment followed by 100 μg/mL Cigarette Smoke Condensate (CSC) for 48 h	RSV at all doses attenuated CSC-induced DNA fragmentation. NPs dramatically increased RSV induced apoptosis in CSC-treated cells	Not provided, but results indicate that NPs are capable of increasing the efficacy of lipophilic drugs such as RSV	[42]
H727	25 μM RSV for 48 h	Decreased cell proliferation and cell viability. Induction of cell cycle arrest	AK001796 a long noncoding RNA (lncRNA) knockdown by resveratrol	[51]
A549	25, 50, 100, 150 μM RSV for 24, 48, 72 h	Inhibition of proliferation in a dose-dependent manner. G0/G1 cell cycle arrest.	(1) Upregulation of p53 nuclear expression (2) Downregulating expression levels of cyclin D1, CDK4, CDK6 (3) Upregulation of p21, p27 which are CDK inhibitors	[44]
A549	50 μM RSV for 12, 24, 48, 72, 96 h	P62 links RSV induced autophagy to apoptosis. P62 inhibits apoptosis by inhibiting Fas/Cav1 complex formation.	(1) RSV degraded P62 allowing Fas/Cav1 complex formation (2) Fas/Cav1 activated caspase-8-mediated Beclin-1 cleavage, resulting in c-terminal Beclin-1 fragment translocation to the mitochondria to initiate apoptosis	[32]
A549	10, 20, 40, 80 μM THS for 12 h	↑apoptosis and autophagy (dose dependent)	↑cleaved PARP ↑caspase-3 and -9 ↓Bcl-2 ↑LC3-II accumulation ↓mTOR pathway ↑ROS levels	[45]
A549	50 μM RSV pre-treatment for 4h followed by H_2_O_2_ treatment (50–1000 μM) with or without RSV for 0.5, 1, 2, 3, 8, 16 and 24h	RSV-loaded nanoparticles restored H_2_O_2_ induced ROS levels	↑RSV uptake ↑Nrf2-Keap1 signalling Accumulation of Nrf2 in abundance	[46]
LLC	1, 2.5, 5, 7.5, 10 μM DHS for 24 h	↓LLC cell growth	↓cell cycle progression ↓cell numbers arresting at G1 accumulation of pre-G1 events correlated with apoptotic behavior ↓LLC cell migration and matrigeal invasion	[47]
CEM and A549	1, 5, 10 and 20 μM for 48 and 72 h	↓proliferation ↑apoptosis	↓tubulin polymerization G2/M cell cycle arrest at 12–18 h period ↓mitochondrial membrane potential ↑caspase-3 and -9, parp-cleavage	[48]

RSV (Resveratrol); PARP (Poly (ADP-ribose) polymerase); LLC (Lewis lung carcinoma); ROS (Reactive oxygen species); NRF-2 (nuclear factor erythroid 2–related factor 2); Bcl-2 (B-cell lymphoma 2); LC3-II (light chain 3-II); mTOR (mechanistic target of rapamycin); CDK4 (Cyclin-dependent kinase 4); CSC (cigarette smoke condensate); H_2_O_2_ (hydrogen peroxide); JNK (Jun N-terminal kinase); SERCA (sarco/endoplasmic reticulum Ca^2+^-ATPase); NSCLC (non-small cell lung cancer); EGFR (Epidermal growth factor receptor); Akt (Protein kinase B); Erk (extracellular-signal-regulated kinase); Gef (Guanine nucleotide exchange factor); EMT (Epithelial–mesenchymal transition); TRAIL (TNF-related apoptosis-inducing ligand); Nox5 (NADPH Oxidase 5); EF1A (Elongation factor 1-alpha); Glut-1 (Glucose transporter 1); SA-B-gal (Senescence-associated beta-galactosidase); HIF-1a (Hypoxia-inducible factor 1-alpha); 18F-FDG (18F-fluorodeoxyglucose); HSP70 (Heat Shock Protein 70); JAK (Janus activated kinase); STAT (signal transducer and activator of transcription); WT (wild type); miR-622 (microRNA-622); NF-KB (nuclear factor-KB); TMS (Trimethoxyl Stilbene); IKB (inhibitor of KB); DHS (Dehydrosilybin); GFP-LC3 (green fluorescent protein-light chain 3); TCDD (tetrachlorodibenzo-p-dioxin); CYP1A1 (cytochrome p450 1A1); PI3K (Phosphoinositide 3-kinase); CDC27 (cell division cycle protein 27); LC3 (light chain 3 protein); chk1 (checkpoint kinase 1); PELP1 (Proline, Glutamate and Leucine Rich Protein 1); ↑ (increase); ↓ (decrease).

**Table 2:** Effects of resveratrol (RSV) in a combination treatment against lung cancer: in vitro studies.

Cancer Cell	Dose/Duration	Findings	Mechanism	Reference
A549 and H460	20 μM RSV for 4 h pre-treatment followed by 2, 4, 6 Gy IR treatment	↑IR-induced cell killing in NSCLC through an apoptosis-independent mechanism	↑% of SA-B-gal positive senescent cells ↑double-stranded DNA breaks	[52]
H-2452	10, 15, 20, 25, 30 μM RSV + 20, 40, 80, 160, 320 nM Clofarabine for 72 h	RSV + Clo decreased Msl-1 protein expression, no effect on Bcl-xL levels. Bcl-xL knockout enhanced RSV + Clo inhibition of cell proliferation and increase in apoptosis	G2/M phase cell cycle arrest ↑caspase-3 and -7 activity and ↑ caspase-3 cleavage	[53]
H460, A549, PC-9 and H1975	5, 10, 15, 20, 40, 50 μM RSV + 1, 2, 4, 6, 8, 10, 12, 16, 20, 32, 40, 80, 160 μM Erlotinib for 24, 48, 72 h	↓cell viability, colony formation and induction of apoptosis. ↑Erl-induced apoptosis	↑ROS production ↓expression of anti-apoptotic proteins, such as survivin and Mcl-1 ↑p53 and PUMA expression ↑capase-3 activity Combination was more effective at inhibiting the Akt/mTOR/p70s6K pathway	[54]
A549 (grown as spheroid bodies to resemble CSC)	ZD55 oncolytic adenovrius carrying the TRAIL gene (ZD55-TRAIL) + 50 μM RSV for 48 h	ZD55-TRAIL alone induced cytotoxicity. Combination of ZD55-TRAIL and RSV increased ZD55-TRAIL mediated cytotoxicity. Apoptosis induction was caspase dependent	↓pro-caspase-9, 8, 3	[51]
PC9/G	1 μM Gefitinib + 40 μM RSV (1) Gef pre-treatment for 24 h followed by RSV for 48 h (2) RSV pre-treatment for 24 h followed by Gef for 48 h (3) Gef + RSV concurrently for 72 h	RSV synergizes with Gef to inhibit the proliferation of Gef-resistant NSCLC cells. Co-treatment induced apoptosis, autophagy, cell cycle arrest and senescence	(1) ↓EGFR phosphorylation by increasing Gef intracellular accumulation (2) ↑CYP1A1 and ABCG2 expression (3) ↑expression of cleaved caspase-3, LC3B-II, p53 and p21	[55]
Lung fibroblast affected by idiopathic pulmonary fibrosis	10, 20, 25, 30, 40, 50, 75, 100, 125, 150, 200 μM RSV pre-treatment for 2 h before treatment with 4 ng/mL TGF-B or 100 pM CXCL12 for 2, 4, 8, 12, 24 and 48 h Or 4 ng/mL TGF-B or 100 pM CXCL12 for 24 h followed by RSV for 24 h	Fibroblast to myofibroblast conversion is reversed and repressed in lung and prostate fibroblasts	(1) RSV 50 μM and below repressed and reversed myofibroblast phenoconversion, but had no effect on N1 or primary prostate fibroblast cell proliferation, apoptosis or COL1 and EGR1 gene transcription. (2) RSV 100 μM and above induced the same effects observed in N1 and primary prostate fibroblast, in IPF lung fibroblasts	[56]

RSV (Resveratrol); CXCL-12 (chemokine (C-X-C motif) ligand 12); COL1 (Collagen Type I); EGR1 (Early Growth Response protein 1); IPF (Idiopathic pulmonary fibrosis); Gef (Guanine nucleotide exchange factor); TGF-B (Transforming growth factor beta); NSCLC (non-small cell lung cancer); CYP1A1 (cytochrome p450 1A1); ABCG2 (ATP-binding cassette sub-family G member 2); LC3B-II (light chain 3 beta-II); EGFR (Epidermal growth factor receptor); TRAIL (TNF-related apoptosis-inducing ligand); ROS (Reactive oxygen species); Erl (Erlotinib); PUMA (p53 upregulated modulator of apoptosis); Akt (Protein kinase B); mTOR (mechanistic target of rapamycin); SA-B-gal (Senescence-associated beta-galactosidase); Mcl-1 (myeloid leukemia cell differentiation protein 1); Bcl-xL (B-cell lymphoma-extra large); Msl-1 (Male Specific Lethal 1); ↑ (increase); ↓ (decrease).

**Table 3:** Effects of resveratrol (RSV) against lung cancer: in vivo studies.

Animal Model	Dose and Duration	Findings	Mechanism	Reference
Female nude mice (5 weeks old) injected s.c. with A549 cells	50 mg/kg DHS i.p. daily from day 1 to 4 and day 7 to 10	↓tumor growth	-	[26]
18 female BALB/c nude mice were injected subcutaneously with SPC-A-1 cells in their flank	Diet supplemented with 1 g/kg/day or 3 g/kg/day resveratrol, for 28 days	↓tumor growth	-	[27]
4–6 weeks old nude were inoculated with A549 cells	20 mg/kg every other day of resveratrol for 25 days	↓metastasis	Activation of SIRT1	[57]
Nude mice subcutaneously injected with A549 cells	15, 30, 60 mg/kg RSV injection for 15 days	↓lung cancer growth in a dose-dependent manner	-	[38]
Male laka mice treated with 100 mg/kg body weight Benzo(a)pyrene to induce lung carcinogenesis	5.7 μg/mL RSV in drinking water + 60 mg/kg body weight curcumin for 22 weeks	BP treatment alone lead to	↑p53 expression and phosphorylation (activation) ↓caspase-3 and -9	[58]
			RSV + Curcumin treatment lead to ↓p53-hyper-phosphorylation and ↑caspase-3 and -9 enzyme activity	
Male C57B6 mice (4 weeks old) bearing LLC tumours	25 mg/kg/day DHS in drinking water for 7 days	↓tumor volume, cell proliferation, tumor angiogenesis and liver metastatic lesions	-	[47]
4–5 weeks old C57B/6 mice injected with LLC	100 mg/kg/day from day 10 until sacrifice at week 4	↓F4/80+ macrophages	M2 macrophage markers (IL-10, Arg1 and CD206)	[59]

RSV (Resveratrol); DHS (Dehydrosilybin); LLC (Lewis lung carcinoma); SIRT1 (silent mating type information regulation 2 homolog 1); BALB/c (Bagg Albino/c); BP (Blood pressure); ↑ (increase); ↓ (decrease).
